# Climate Change and Food Security: A Framework for Agenda Setting and Policy Analysis in Iran

**DOI:** 10.3389/ijph.2025.1608116

**Published:** 2025-04-16

**Authors:** Ramesh Allipour Birgani, Amirhossein Takian, Ali Kianirad, Hamed Pouraram, Abolghasem Djazayeri

**Affiliations:** ^1^ Department of Community Nutrition, School of Nutritional Sciences and Dietetics, Tehran University of Medical Sciences, Tehran, Iran; ^2^ Center of Excellence for Global Health (CEGH), Department of Global Health and Public Policy, School of Public Health, Tehran University of Medical Sciences (TUMS), Tehran, Iran; ^3^ Department Health Management, Policy & Economics, School of Public Health, Tehran University of Medical Sciences, Tehran, Iran; ^4^ Health Equity Research Center (HERC), Tehran University of Medical Sciences, Tehran, Iran; ^5^ Agricultural Planning, Economic and Rural Development Research Institute (APERDRI), Tehran, Iran

**Keywords:** agenda setting, climate change, policy analysis, food security, food policy

## Abstract

**Objectives:**

This study identifies the key factors contributing to Food Security (FS) in the context of Climate change (CC), aiming to foster agenda setting for FS in Iran.

**Methods:**

This is a qualitative study. We interviewed 32 relevant stakeholders from various backgrounds. We used a mixed inductive–deductive approach in data analysis, drawing up on an adopted framework comprising of health policy triangle and selected agenda setting framework.

**Results:**

Our analysis revealed eight constructs, eight themes, and 26 subthemes. The constructs included: common voice, leadership, scientific evidence, economic, multi-sectoral collaboration, advocacy, early warning systems, and supreme decision-making center. The main themes identified were shortcomings in: consensus, high-level political commitment, cooperation, System approach, research, planning, economic resources, and public participation. The international data gathering was limited in this study.

**Conclusion:**

To mitigate the risk of FS in CC condition and push the emerging subject into the government agenda in Iran, we recommend reforms in the eight identified constructs and advocate a combined policy approach including three dimensions: policy integration, coherency, and coordination, through a new model of governance.

## Introduction

Climate change and food security are two wicked problems that influenced the environment, ecology, economic, health, and social dimensions of nations’ life [1]. These climate change (CC) events have influenced four components (food availability, food accessibility, food utilization, food sustainability, and stability) of food security (FS) [2]. It means that agricultural food production, food affordability and food prices, food safety, accessibility to water, oil prices, energy prices, health cost, population growth rate, and income of people that live in these areas have affected and threatened [3]. However, 25%–30% of Green House Gas (GHG) production is relegated to agricultural production [4]. Most areas in the globe are endangered more than others by whether extreme events. CRED (Center for Research on The Epidemiology of Disaster) reported in 2022, droughts impacted 88.9 million people in six African countries (the Democratic Republic of the Congo, Ethiopia, Nigeria, Sudan, Niger, and Burkina Faso) and induced famine in Uganda causing 2,465 deaths. The cost of damaged by drought events occurred in three countries (China, USA, Brazil) worth 33.6 billion US$ in 2022. Hurricane “Ian” caused damage costing 100 billion US$ in the Americas. The economic damage of floods only in 2022 in countries (Pakistan, India, China, Nigeria, and Eastern Australia worth 34.6 billion US$ [5]. Moreover, global warming and the increased frequency and severity of climate extreme events are responsible for malnutrition and affected 422 million population in low-income countries [6]. Evidences revealed that, 30 countries in Africa and Asia which produce 80% of the crop by self-sufficiency are vulnerable to CC, hence, their people are more susceptible to hunger, poverty and are at risk of malnutrition. In Pakistan in the food-insecure provinces of Baluchistan and Sindh between June and August 2022, more than 11 million heads of livestock were killed by floods, and more than 9.4 million acres of cropland were destroyed [7]. The Middle East with 22 countries, is the region where the population experienced drought, flood, sand, and dust storms [8]. Iran is located in this region and is one of the ten most disastrous countries [9]. The precipitation is one-third compared to the mean world precipitation and the temperature increases 0.5 C (centigrade) every decade during the last three decades [10]. In addition, 70% of groundwater sources would with throw, and water bankruptcy is distinguished [9]. Two third of the climate in this country is arid and semi-arid [11], further, for three decades a large number of the population experienced drought and flood [12]. In 2019, 27 out of 31 provinces had affected by floods due to 2.5 billion dollars lost. A million hectares of agricultural land and production were damaged in these areas [13]. Iran is known as the seventh GHG producer among 187 countries in the 2020 report because a substantial part of countries’ GDP is reforming oil and fossil fuel exportation [14]. The considerable point is, population growth policies and agricultural food production self-sufficiency which is followed by the government. Accordingly, policy analysis of Iran’s FS under CC was necessary to understand key leverages, problems, and policies. Several studies demonstrated that the policy model was helpful to policy analysis of health, FS, and CC, e.g., the “Shiffman framework,” with four components (Actor Power, Ideas, Political Context, and Issue characteristics) [15–17]. In Iran, there is a scarcity of studies investigating this topic, leaving a knowledge gap. Thus, we applied the “Shiffman framework” to policy analysis of FS under CC to introduce a governance model of food policy in Iran [18]. Such needed studies might inform decision-makers in the country about the impact of CC on FS and would be helpful to push this issue in agenda setting.

## Methods

**FIGURE 1:**
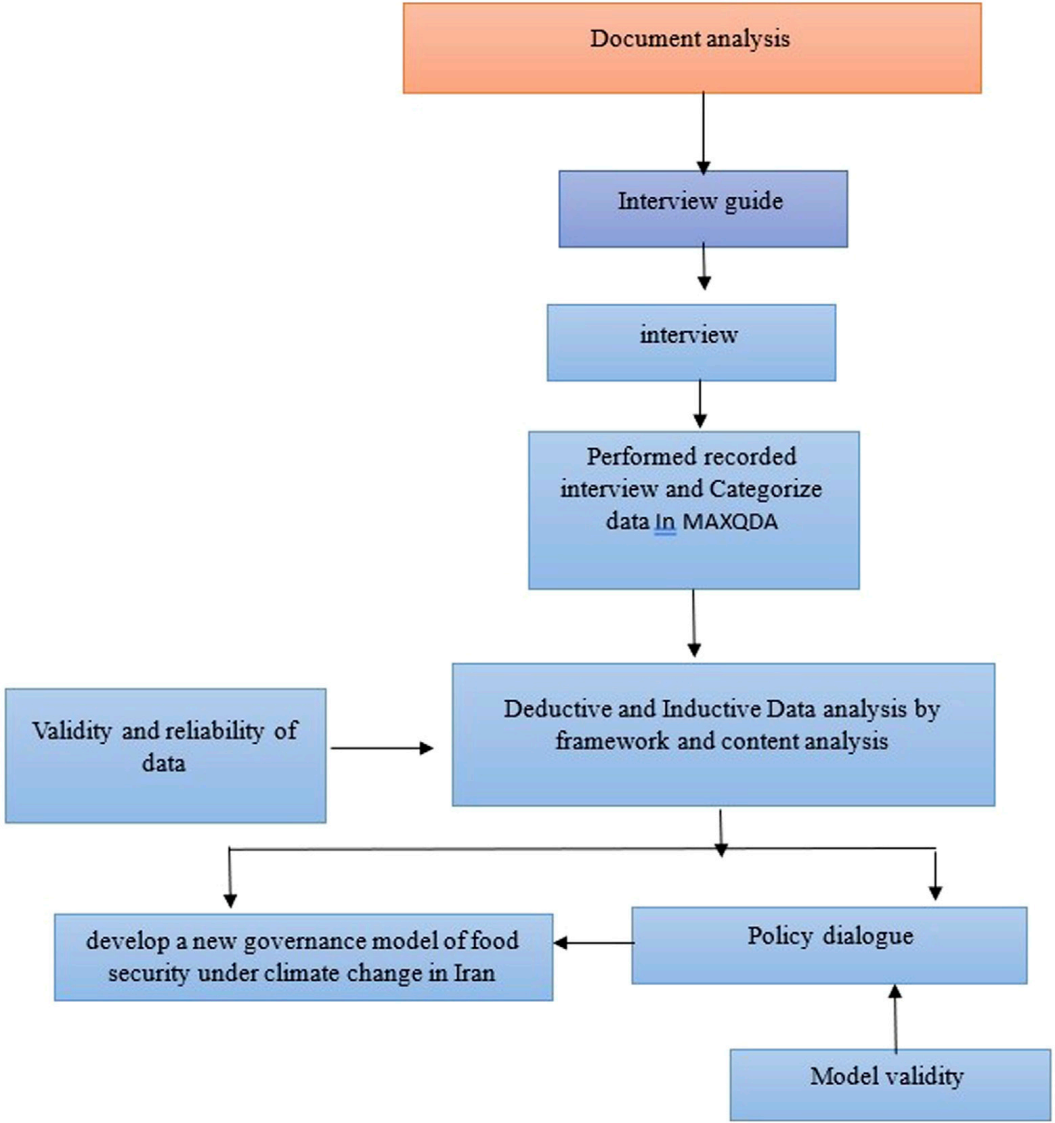
The conceptual framework of the qualitative study (The study of Climate Change and Food Security policy analysis, Iran, 2022).

This is a qualitative study. We used three methods of data collection for policy analysis: document analysis and semi structured expert interviews [19]. In the first stage of the qualitative policy analysis study, we applied framework analysis and then used a deductive approach according to four FS components and CC (three disaster risk factors: exposure, hazards, and vulnerability) [20, 21]. Afterward, through document analysis and interviews, we applied an inductive approach to identify the main structures of policies [22]. In the third stage, we applied two frameworks, the “Shiffman framework and triangle policy,” to inform the interview design, data analysis and interpretation of the research findings [18, 23]. After extracting the codes and constructing the themes, we used the themes to develop the constructs and to design a new governance model of FS under CC for Iran. Policy dialog was performed for the validity assessment of the new model [24]. Twelve key informants took part in policy dialog and presented their ideas about vertical and horizontal positions and the relationships among the constructs in the governance model. The conceptual framework of the study methodology is presented in Figure 1.

### Data Collection and Sampling

We selected 32 experts from 30 official and unofficial organizations, institutes, and private sectors related to FS and CC in national policy-making in Iran via purposive sampling and snowball sampling from April 2019 to May 2020 [25, 26]. However, we continued sampling until saturation occurred. That is to say, there was no new theme or insight to identified and explored from data collection during interview by researchers. The inclusion criterion for sampling was at least 5 years of experience in related issues (FS and CC) in academic or executive positions. To achieve greater sampling validity and provide different policy options, we selected samples with acceptable disparities from all related organizations with maximum variation. Table 1 summarizes the list of interviewees.

**TABLE 1:** The list and characteristics of interviewees (The study of Climate Change and Food Security policy analysis, Iran, 2022).

Experience (year)	Sex	Education	Workplace of interviewee (abbreviation)	Code Person (P)
30	F	PhD	MoHME	P1
31	M	PhD	IKRF	P2
25	F	MSc	DoE	P3
28	M	MSc	MoA	P4
8	M	PhD	MoA	P5
55	M	PhD	MoHME	P6
15	F	PhD	IRIMO	P7
25	M	PhD	MoA	P8
34	M	PhD	MoHME	P9
18	F	MSc	MIMT	P10
16	M	PhD	(Food & drug organization) MoHME	P11
35	M	PhD	(Industrial food and nutrition institute of research) MoHME	P12
15	M	MSc	MoHME	P13
15	M	MSc	MoA	P14
45	M	PhD	MoHME	P15
5	M	PhD	MRUD	P16
18	F	PhD	MCLSW	P17
25	M	PhD	APDIM	P18
28	M	PhD	(The forestation organization) MoA	P19
30	F	PhD	(Faculty of nutrition) MoHME	P20
28	M	PhD	(The research institute of agricultural economic) MoA	P21
35	M	PhD	PBO	P22
25	M	PhD	(The environmental department) MoA	P23
26	F	PhD	MoE	P24
32	M	MSc	MFA	P25
29	F	MSc	MoHME	P26
25	M	PhD	MoA	P27
32	F	MSc	MoP	P28
29	M	PhD	MoA	P29
25	M	PhD	MoHME	P30
30	M	PhD	MoA	P31
20	M	PhD	MoI	P32

Note: M, Male; F, Female; IRIMO, Islamic Republic of Iran Meteorological Organization; NDMO, National Disaster Management Organization; MoA, Ministry of Agriculture; MoHME, all data extracted from food balance sheet, Ministry of Health and Medical Education; MIMT, Ministry of Industry, Mine, and Trade; NCoS, National Center of Statistics; MoE, Ministry of Energy; MoP, Ministry of Petroleum; DoE, Department of Environment; MFA, Ministry of Foreign Affairs; MoI, Ministry of Information; PBO, Planning and Budget Organization; APDIM, Asian and Pacific Centre for the Development of Disaster Information Management; MRUD, Ministry of Roads and URBAN Developments; MCLSW, Ministry of Cooperatives Labor and Social Welfare; IKRF, Imam Khomeini Relief Foundation.

We obtained 41 national and international documents, reports, and parliamentary proceedings related to FS and CC through a literature review and carried out document analysis according to a directed and conventional content analysis framework to extract codes, themes, and subthemes [20, 27].

### Interviews

We conducted semi structured in-depth and face-to-face interviews aimed at exploring the principal governance issues of FS and CC policies in Iran. [25]. We scheduled interviews based on policy analysis frameworks (Shiffman framework; FS dimensions, health policy triangle framework) [18, 23] and designed an interview guide with 13 questions. In this stage, the trained researcher asked for information on actors’ power and influential stakeholders; policy problems and characteristics; policy ideas and priorities; policy and political context; framing of FS and CC (policy process); opportunities to improve governance weaknesses; and threats to policy making from interviewees. Then, these two policy frameworks were used for data analysis and assisted interpretation of the research findings. Furthermore, based on the four stages (agenda setting, formulation, implementation, and evaluation) of the policy process, the codes were extracted [28]. We analyzed the informants’ interview data to identify key themes and subthemes related to food and nutrition policy. Every interview lasted 45–90 min. The interviews were recorded digitally and transcribed verbatim. Before the interview, the researcher sent an information sheet about the purpose of the study and a consent form to reassure the interviewees about anonymity, freedom to withdraw, and approval of participation by completing the informed consent form.

### Data Analysis

**FIGURE 2:**
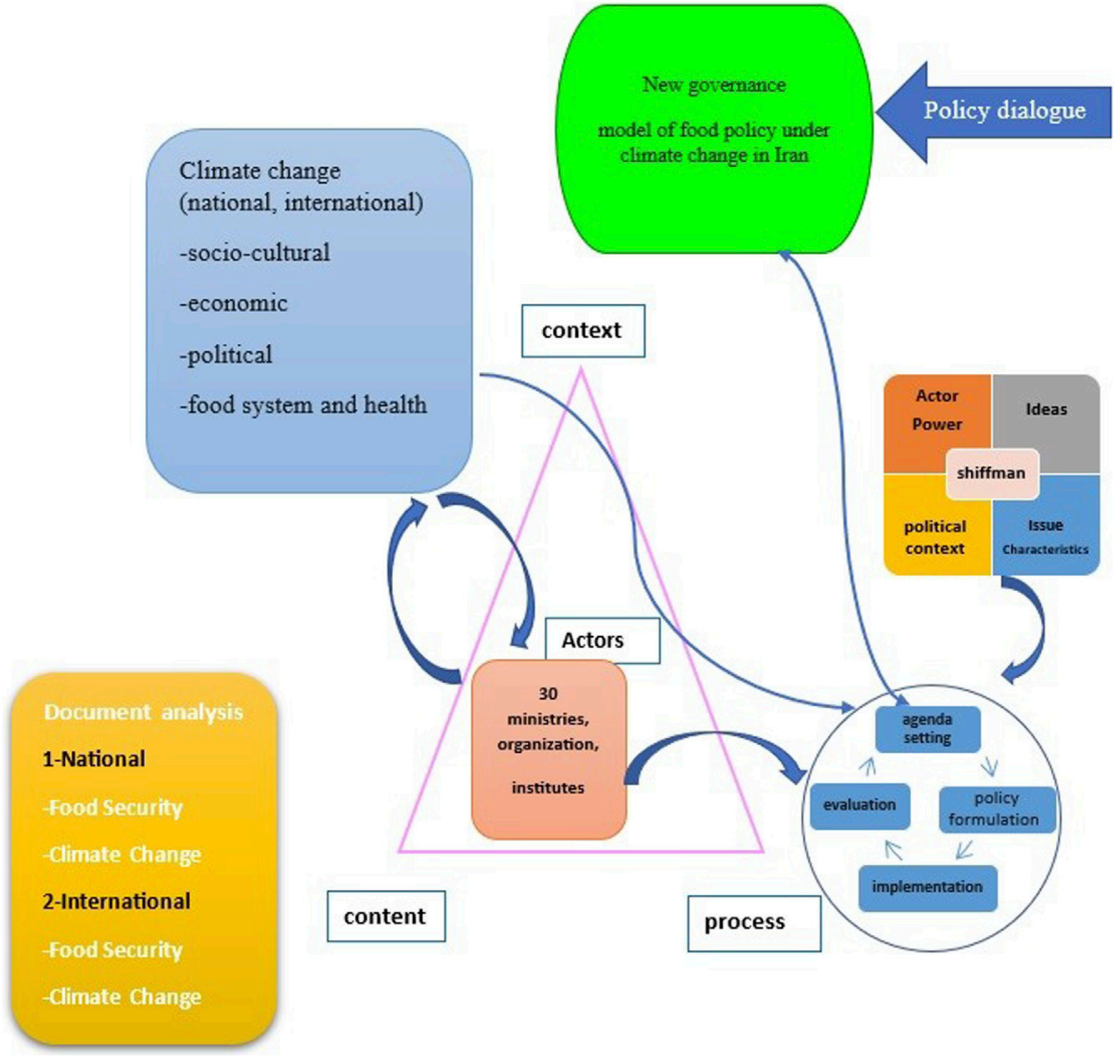
The conceptual framework of policy analysis according to the health policy triangle and Shiffman and Smith policy framework (The study of Climate Change and Food Security policy analysis, Iran, 2022).

We utilized framework analysis to analyze the data interviews in five phases: Familiarization, Identifying a thematic framework, Indexing, Charting, Mapping, and Interpretation [20]. We used MAXQDA software version 2020 for data management. We applied a mixed deductive-inductive approach, for thematic analysis. The deductive approach was utilized to identify codes according to FS dimensions and CC disaster risk factors. Then, based on an inductive approach, conventional content analysis was used to find new and latent codes and to develop themes and subthemes. The first and corresponding authors carried out the categorization process, and the other authors revised and approved the entire process. The validity and reliability of the findings were assessed by the methods of Guba and Lincoln [29]. We invited two researchers to assess the external validity of the coding system. The conceptual framework of policy analysis according to the health policy triangle and Shiffman policy framework is presented in Figure 2.

## Results

The identified themes, subthemes, and constructs (8 themes, 26 subthemes, and 8 constructs) related to FS under CC government policy in Iran are summarized and reported in Table 2.

**TABLE 2:** The identified themes, sub-themes, and constructs identified according to Shiffman framework related to food security under climate change government policy making (Iran, 2022). (The study of Climate Change and Food Security policy analysis, Iran, 2022).

Sub-themes	Themes	Constructs	Shiffman framework components
1. Legislation 2. Decision making 3. Conflicting interest 4. Conflict of interest	System approach	Supreme Decision-making center	Political context
1. Indicators 2. Risk assessment & needs assessment 3. Foresight 4. Long run planning 5. Monitoring & evaluation	Planning	early warning system	Issue characteristics
1. Ignore evidence 2. Multidisciplinary research 3. Limited evidence	Research	Scientific Evidence	Issue characteristics
Executive Guarantee	High-level political commitment	Leadership	Political context, Actor power
Cost Benefit	Economic support, financing	Economic evaluation	Political context Issue characteristics
1. Common goals 2. Priority selection 3. Stakeholders’ beliefs	Consensus	Common voice	Ideas
1. International collaboration 2. National solidarity	Cooperation	Multi-sectoral collaboration	Political context Actor Power
1. Community education 2. Lobbing 3. Capacity building	Public Participation	Advocacy	Actor power

**FIGURE 3:**
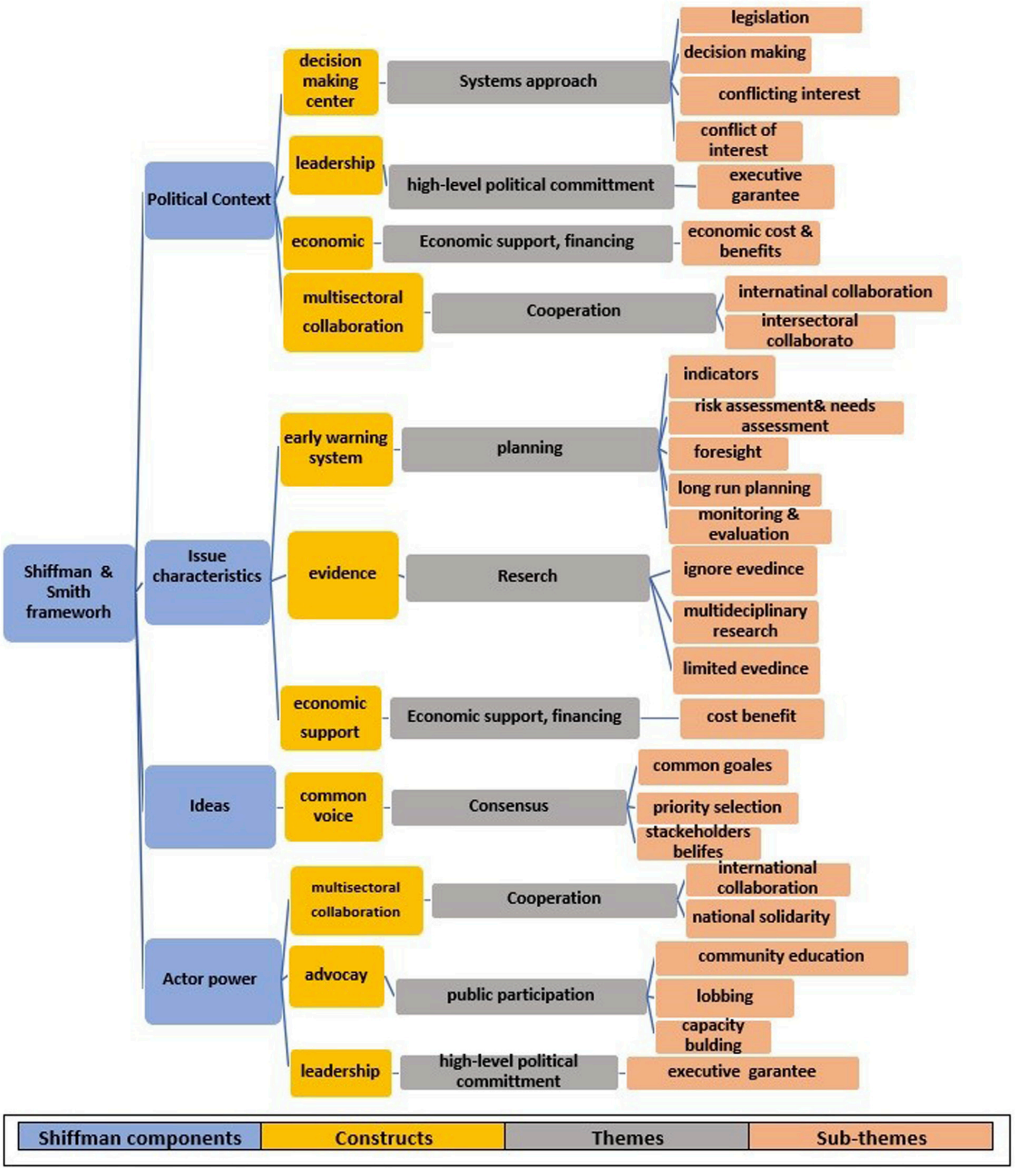
Tree framework of sub-themes, themes and constructs of food security under climate change policy analysis in Iran based on “Shiffman and Smith” policy framework (The study of Climate Change and Food Security policy analysis, Iran, 2022). Note: sub-themes were investigated based on the codes that identified with food security dimensions availability, accessibility, utilization, stability or sustainability) and climate change disaster risk factors (exposure, hazards, and vulnerability), then themes and constructs were explored and categorized according to “Shiffman and Smith” policy framework with four components (Actor Power, Ideas, Political Context, and Issue characteristics) and policy triangle with three dimensions (context, content, process).

The results of the data analysis according to the 8 explored themes and subthemes are presented with evidence obtained from the key informants’ comments in the following paragraphs. The results are presented in Figure 3.

In this framework, all of the constructs, themes, and subthemes extracted based on threats and barriers to agenda setting for FS under CC in the context of Iran are presented. Several pieces of evidence explored from informants’ interviews are reported through cotes in the next paragraph. These cotes are categorized according to the components of the Shiffman health policy framework [18].

### Political Context

We addressed 821 codes in this component and then categorized them into 9 subthemes and 4 themes. Finally, we developed 4 constructs. A weak system approach was a consequence of poor legislation, weak decision-making, conflict of interests, and conflicting interests. Several informants noted that the weakness or lack of legislation or overlooking stakeholders and decision-makers in the legislation process, the scarcity of evidence for legislation, and the contradiction of laws in FS under CC in Iran were the greatest barriers to appropriate legislation. An informant stated that a shortage of legislation is a crucial

*“There is a problem in addressing a big picture of CC in the country, as well as national legislation in mitigation aims to decrease fossil fuel use as energy. The parliament ought to enact a law for using renewable energy and CC mitigation and adaptation strategies, while there is limited governmental policy making to deal with these complicated problems and risks.*” (p 13)

*Some informants complain about no executable FS and CC laws. One of them expressed that “from 2002 there are a couple of enactments in the supreme council of Health and FS that never executed. This is because there was no will for professional activity based on the scientific backbone for providing these laws. Hence, there are no administrations.”* (P 1)

### Issue Characteristics

This component of the Shiffman framework describes the problem’s picture [18]. In this study, after the interview analysis, we extracted 1148 codes, categorized them into subthemes, and distinguished three themes (programming, research, and finance or economic support). Ultimately, we created three constructs, which are presented in Table 2 and Figure 3. In the next paragraphs, to understand a different point of view, we present some cotes related to different problems of FS under CC in Iran.

*Some interviewees believed that there was a lack of indicators, limited risk assessment and needs assessment, foresight scarcity, uncommon long-term planning, and weak monitoring and evaluation due to defective planning. One of the experts highlighted the estimation of aims:* *“We do not have quantitative estimation of aims in our action plans. Therefore, we could not address our needs, legislation or decision making based on rigorous aims. For example, if we decide to diminish 0.5% of malnutrition in children in our country, it is necessary to accurately investigate this disease and design quantitative targets.” *(P 5)

*A number of experts illustrated the connection between exposure and risk assessment in national FS planning under CC: “We have to know how many people in our country are exposed to risks of food insecurity. Because for national food insecurity mapping and estimation the extent of exposure to this threat, formulation of risk reduction based on categorization of the variables into external, internal, local and regional is indispensable.”* (P 18)

### Ideas

In this component of the Shiffman framework [18], we identified 220 codes and categorized them into three subthemes (common goals, priority setting, and stakeholders’ ideas). Then, a consensus theme was created, which led us to develop a construct named the common voice. The experts stated that a common viewpoint is necessary to reach a consensus on FS under CC. This persuades policymakers to put this subject on the agenda. In the next paragraphs, some cotes are presented to better highlight this issue.

*An informant illustrated the lack of common ideas: “There is no opportunity to solve the CC and FS problems in our country until we believe in working lonely and in our islands without any consensus on common priorities”.* (P 1)

Other informants explained the role of the Supreme Council of Health and Food Security (SCHFS) in performing common ideas:

*“The role of this organization is conducting multi-sectoral collaboration among all stakeholders that contributed to health issues. The chief of the SCHFS is the president, and the health minister is the secretary, but unfortunately, there was not enough success in performing common ideas to push FS and CC issues on the agenda. This is because some decision makers suppose that heath is a luxury subject. Hence, these subjects are in second or third rank.”* (P 13)

Some experts noted the importance of stakeholders’ beliefs on common ideas for FS under CC agenda setting in Iran:

An informant explained the substantial role of decision making in CC management*:*

*“In Iran, most policymakers have made efforts toward disaster management to control the harmful impacts of CC, although they have to pay attention to risk management to prevent the major negative impacts of this issue on people and the environment and decrease vulnerability”.* (P 31)

### Actor Power

The fourth component of the Shiffman framework is actor power [18]. We identified 314 codes and categorized them into six subthemes (international collaboration, community education, national solidarity, capacity building, executive guarantees and lobbing). Three themes were subsequently identified (national determinants, Public Participation and cooperation). Below, we present the viewpoints of the informants about these subjects.

Community education: Some interviewees believed that community partnership is a valuable capacity for FS and CC community education as well as for advocacy. People are powerful stakeholders.

The informant noted the substantial role of public participation, adaptation and mitigation policy education*:*

*“People mute aware about the considerable role of their activities in declining the harmful impacts of CC on food resources. Moreover, the acculturalization of food production and consumption is essential for decreasing GHG emissions and diminishing resources, e.g., soil erosion and water misuse. Are people aware of these problems? Are the government involved in collaboration in education, forestation and pastural landscape expansion?”.* (P 27)

Public participation: A number of experts stated that community education, lobbying and advocacy are important. Therefore, the government could apply this opportunity to increase FS under CC in Iran.

*An expert illustrated the capacity of people to develop FS under CC. “If we informed our people about the harmful effects of deforestation and changed pastures to agricultural land for more food production, we would never be able to confront this extent of drought and pasture damage. This is because we did not use community participation’s capacity and environmental acculturalization to improve people’s information and behaviors.”*

### National Governance Model of Food Security Under Climate Change

**FIGURE 4:**
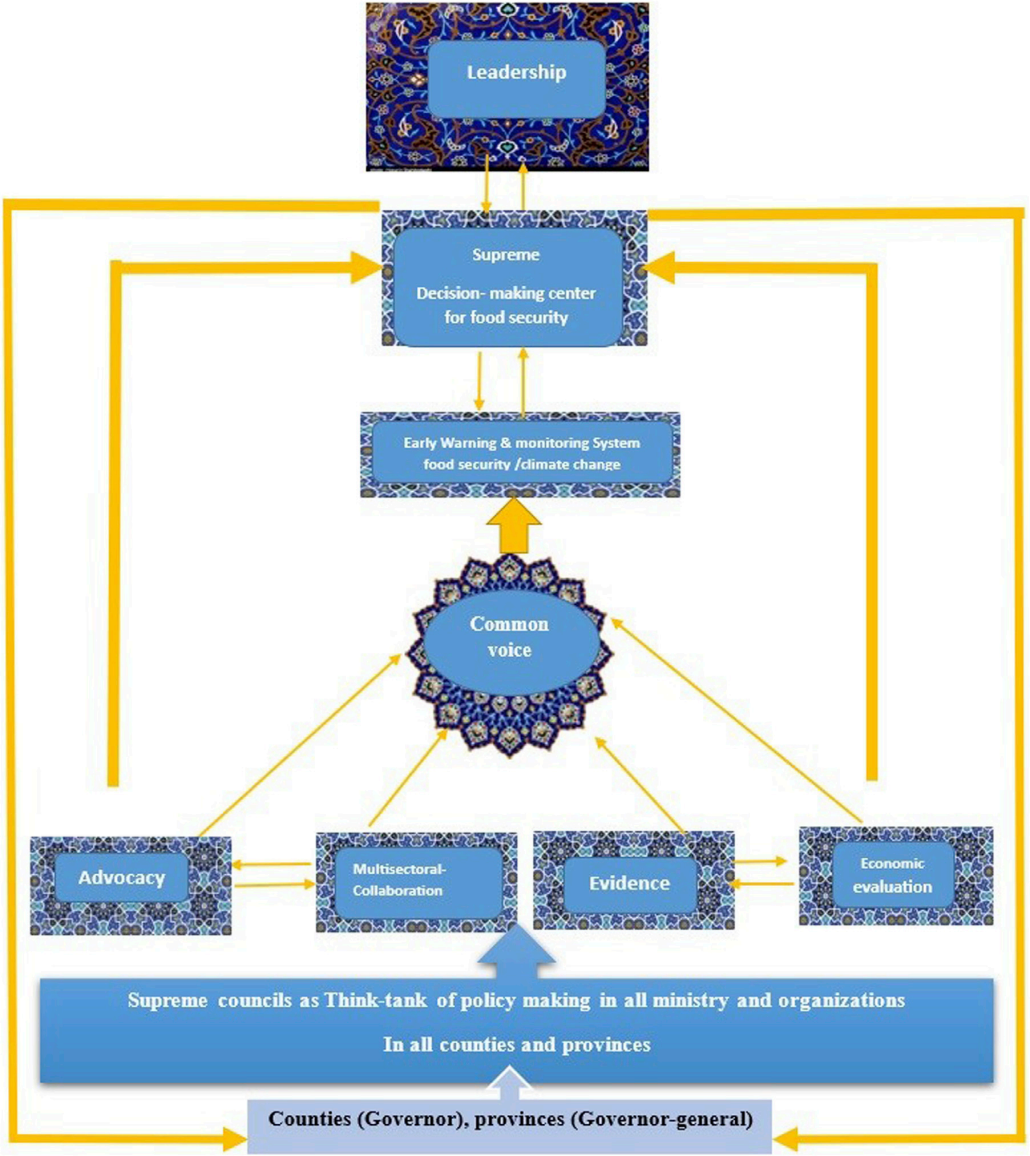
Model for governance food security and climate change policy -making (The study of Climate Change and Food Security policy analysis, Iran, 2022).

Based on 8 constructs explored from the themes, we developed a model of FS under CC for the governance policy-making in Iran. Find it in Figure 4.

## Discussion

In this study, policy analysis based on the four components of Shiffman’s policy framework highlighted a critical issue for (FS) and (CC) agenda setting [18]. It has revealed that certain barriers play a detrimental role in impeding effective decision-making for the implementation of national (FS) and (CC) programs: lack of (food decision-making center, leadership, economic support, multi-sectoral collaboration, early warning system, evidence, common voice, advocacy).

One fundamental problem is the governance fragmentation of FS, which leads to some issues in decision making by various institutes and organizations. This is partially due to conflicts of interest and divergent interests. Moreover, these barriers have a significant impact on the development of fair and equitable legislation. This complex situation arises due to the utilization of powers as non-decision-making by some stakeholders that employ powers to obstruct the creation of impartial legislation. However, the evidence showed that many countries face the same situation [30–32].

Another issue is parallel and conflicting legislation, Our finding indicates, this factor poses significant barriers to consensus on FS under CC national policy agenda setting in Iran, e.g., three key stakeholders namely, the Food and Drug Administration (FDA), the International Institute of Standards, and the Ministry of Agriculture are tasked with setting standards for assessing the levels of chemical contaminants and evaluating pesticide or herbicide residues in agricultural food products with different standards, that confused farmers [33–35]. This behavior come from economic interests of organizations [36]. Therefore, policymakers must prioritize policies that align with the shared objectives of all stakeholders aim to push the policy on agendas [36].

Addressing CC is a global imperative, necessitating international collaboration. Consequently, the Paris agreement was established to convene all nations around a common goal of reducing global warming [37]. This study indicated that, lack of inter-sectoral collaboration and international cooperation, stemming from weak multi-sectoral collaboration within the political context of Iran are significant barriers [38, 39].

Furthermore, several “issue characteristics” emerged from the subthemes, highlighting the importance of specific factors: limited indicators, risk assessment, needs assessment, long-term planning, monitoring and evaluating the impacts of CC on FS in Iran, and cost‒benefit considerations for FS programs. Consequently, the establishment of an early warning system to monitor FS indicators and to provide accurate projections which show the severity of the situation is essential to informed FS policy-making under CC within the governance framework.

A feature of weak inter-sectoral collaboration is come from lack of effective data sharing by various institutions, organizations that diminished their ability to plan for the future based on accurate evidence, transparency, and accountability. Consequently, the adaptation programs related to CC and FS were unsuccessful, primarily because all stakeholders failed to fulfill their roles and responsibilities in shaping, planning, and implementing the policies. The other disadvantage of weak inter-sectoral collaboration is susceptibility of FS in high-risk communities to CC risks. The evidence from studies in other countries supported our results [40–42]. For instance, during the extreme flood events in 2017–2018 and 2020–2021 in some provinces in Iran, all four dimensions of FS were jeopardized, and many residents in these regions faced different features of food insecurity [43–45].

To shed light on the successful experience, Sweden’s decision makers employed a policy convergence approach at the highest levels of government and created a new environmental policy framework to effectively implement a policy with 15 well-defined objectives and the active involvement of 24 government institutions and entities [46]. Their approach to addressing inter-sectoral collaboration challenges and mitigating conflicts of interest through policy integration aligns closely with our research that introduces a new governance model focused on policy integration to achieve FS under CC in Iran. This model takes into account the diverse powers, ideas, and political contexts of the various involved actors. In addition, one crucial factor contributing to the prioritization of a subject is achieving consensus [47, 48]. In the current study, stakeholders engaged in the examination of FS within the context of CC encountered challenges in establishing a consensus. Accordingly, they struggle to underscore the significance of this particular subject matter, substantiated with adequate evidence, for high-level policymakers. As such, in accordance with the Shiffman model and the structural component of “Ideas”, common voice building is advocated as a foundational solution in this context [18].

The other identified obstacles for FS under the CC agenda setting in Iran is the lack of interaction, societal participation, and high-level political commitment, which is closely tied to the influence of various actors at different levels of government and within the government and private sectors related to FS and CC [49]. Also indicated an imbalance in the distribution of power among different actors. Therefore, within the framework of the agenda setting framework, the component of “actor power” suggests strategies for inter-sectoral collaboration, advocacy, and leadership to engage civil society in exerting pressure on these groups to steer policies toward prioritizing the issue.

To ensure FS in Iran and address the critical aspect of agenda setting within the nation’s overarching policy framework, bridging the gap between policy objectives and their practical implementation is essential. Thus, presenting an evidence-based approach, one that considers the identified structures to map out interactions among diverse stakeholders during the policy-making process, is imperative [48]. Consequently, drawing upon best practices from various nations, this study introduces a comprehensive model for governance and policymaking in the realm of FS, particularly under the influence of CC conditions in Iran. This model is visualized in Figure 4.

The basis for policy convergence involves factors such as social behaviors, institutional coalitions, and the effectiveness of institutional policies. Moreover, the relationship between subsystems. It can lead to the creation of a network of cooperation to address issues within the government structure, thus offering opportunities for horizontal collaborative solutions [48].

In fact, the result of such a process can give rise to a new subsystem within the government structure. The results of this study have shown the effectiveness of this framework in placing two policy issues, food policies and CC adaptation, on the agenda. Since CC affects policies in all sectors, it is necessary to develop plans that incorporate policy convergence in adaptation policies, such as the Paris Climate Agreement and Sustainable Development Goals [48, 50].

In the present study, considering the identified challenges, the political context of the country, the power of stakeholders, and the absence of a common voice, it appears essential to have an institution capable of making decisions beyond the established boundaries between ministries and agencies. This institution should possess a broad perspective on governance levels and hold legitimate authority in legislation and decision-making in the realms of FS and CC.

In such an environment, the possibility of formulating policies and making decisions with minimal opposition, conflicts, and maximum agreement and saliency is facilitated. Furthermore, the necessary conditions for supporting various stakeholders at different levels are established.

In successful experience, the Netherlands employed the policy integration framework method to explore both policy convergence and divergence within different governance structures on FS and CC. [48]. The results of this study were consistent with our findings.

Another effective policy integration framework was established in 2013, when Mexico launched a comprehensive program to combat poverty and hunger at the national level. One program, the “National Crusade against Hunger,” focused on hunger reduction and involved 19 institutions to coordinate efforts. The program aimed to enhance coherence among social programs and improve policy integration. An inter-ministerial communication committee was formed for program selection, budget allocation, and monitoring. The former Mexican President established ministry committees at the national, state, and municipal levels. He was the leader of this committee. It was a successful program to diminish poverty and hunger after 3 decades in Mexico. Their approach of integrating policies and proposing a government model to overcome barriers (weak cooperation, leadership, high-level political commitment, common voice, public participation) in implementing FS policies aligns with the findings and recommended model of FS governance in our study [51].

In recent decades, many countries have globally embraced governmental fragmentation as a strategy to address evolving challenges in public services and the distribution of essential goods. However, this strategy has its drawbacks: it often neglects horizontal collaboration among institutions, leading to a decrease in the central authority of senior managers. Consequently, their impact and effectiveness in local governance declined due to power decentralization. This shift also decreased cooperation between sectors and generated tensions when complex issues related to public services were resolved. According to Peters, the specialization and lack of convergence only give rise to new problems, particularly in complex matters, as a result of dividing government activities [47, 52].

A study in England revealed that the government addressed air pollution from public transportation and rising carbon emissions by adopting a policy integration approach. Facilitated a horizontal coordination among entities and initiate vertical alignment. [53]. The UK government’s strategy of policy integration and prevention of competition among different organizations for funding aligns with our study.

The experience in the Netherlands, aimed at improving FS and overcoming fragmented policy-making while maintaining and enhancing the agriculture, economy, and environmental infrastructure sectors, was designed in collaboration with all sectors and civil society by the Ministry of Economic Affairs and presented to the parliament. This initiative proposed a shift from the Common Agriculture Policy (CAP) to the Common Food Policy, and its draft was supported by think tanks and civil society organizations in Europe [48]. A similar approach is also influential in Iran, emphasizing self-sufficiency in food policies, with the Ministry of Agriculture [54].

Another policy designed in the Netherlands to adapt to CC involved the proposal to merge three ministries: The Ministry of Transport, the Ministry of Water, and the Ministry of Housing to create a new ministry called the Ministry of Infrastructure and Environment according to an integrated policy approach [48].

However, in Iran, CC has led to decreased precipitation and prolonged droughts, coupled with the utilization of seventy percent of underground water resources [55]. Additionally, reactionary and short-term policies such as the construction of dams and the redirection of water flow in some provinces have created water stress [56]. As a result, not only agriculture and sustainable food production are threatened on small farms, but the future of drinking water and industrial water supplies is also a cause for concern [57, 58].

Moreover, approximately two decades ago, Iran established the SCHFS, which aimed to address critical issues related to health and FS. However, despite its establishment, this framework fell short of achieving the desired level of inter-sectoral collaboration among the different actors involved.

Thus, we recommend the establishment of a supreme decision-making body for ensuring FS in the face of CC, with the president serving as its leader. This pivotal body comprises various key stakeholders, including all ministers, the Chief of the Planning and Budget Organization, the Department of Environment, the Speaker of the Parliament, the Chief Justice of the country, and representatives from relevant nongovernmental organizations (NGOs). This central authority would be vested with significant powers to execute comprehensive FS initiatives at both the federal and local government levels, in the vertical (from the federal center to provinces and counties) and horizontal (inter-sectoral collaboration by the activity of the supreme committee of health and FS and other related supreme committees such as Think-Tank) levels of government and using a combination of bottom-up and top-down policy-making. Presented in Figure 4.

Malta’s experience underscored the challenges faced by agriculture due to water scarcity, prompting a renewed focus on FS. A solution was to establish a joint committee among ministries, known as the “cross-ministerial water committee.” Its primary objective was to facilitate vertical coordination across all levels of governance and promote horizontal collaboration among institutions, ministries, and all stakeholders engaged in water-related matters [30]. The findings of this study provide compelling evidence that supports our research.

Another example of successful policy convergence was the implementation of a nexus approach in Brazil, which integrated the energy, food, water, and CC sectors. This experience demonstrated the effectiveness of networking and policy convergence in addressing complex challenges. In 2016, Brazil developed a National Climate Change Adaptation Program, emphasizing the need for coherence with other national programs [40].

According to Hall’s model, issues in the agenda must possess three characteristics: legitimacy, feasibility, and support [59]. Based on the current management model of FS in the context of CC in Iran, as discussed in the previous sections, The performance of SCHFS and other similar councils, numbering more than a hundred and five in the governance system of the Islamic Republic of Iran runs parallel to related ministries [60]. These councils represent clear examples of power division and fragmentation of management, as evidence has shown that their performance has failed to promote collaboration between sectors and policy convergence [61]. In our research, we utilized the Shiffman policy framework analysis as a foundational approach, carefully categorizing our results within this framework. However, we did not stop at mere categorization; instead, we effort to develop a new governance model according to joined-up policy. Moreover, it is designed in a way that, through policy-making based on the coalition of the two approaches, top-down and bottom-up, according to the advocacy coalition framework, engages civil society participation and creates conditions with advocacy, influencing policymakers.

### Conclusion

Achieving a balanced distribution of power and influence among various actors, within a new governance framework for FS under the challenges of CC based on joined-up policy is critical. It encourages all stakeholders to actively engage in a coordinated policy approach. In this restructured framework, each stakeholder considers the interests of all parties involved, conducts comprehensive research, economic cost‒benefit analyses, shares data for monitoring key indicators, and sets priorities aligned with collective objectives. Effective interaction and collaboration ensure the participation of the community and inter-sectoral cooperation with a common voice. These efforts culminated in a central hub for comprehensive FS management, ensuring a unified and effective response “political will” to the challenges posed by CC.

### Limitations

This study focused primarily on the national perspective of FS stakeholders, primarily engaging with national-level policymakers. However, it is important to note that it did not encompass the international viewpoint of influential organizations such as the Food and Agriculture Organization (FAO) or the United Nations Children’s Fund (UNICEF), which are significant players in the global FS landscape. Future research efforts could consider expanding their scope to involve international organizations such as the FAO or UNICEF.
